# Polygenic Risk, Modifiable Lifestyle Behaviors, and Metabolic Factors: Associations with HDL-C, Triglyceride Levels, and Cardiovascular Risk

**DOI:** 10.3390/nu17132244

**Published:** 2025-07-07

**Authors:** Danyel Chermon, Ruth Birk

**Affiliations:** Nutrition Department, Health Sciences Faculty, Ariel University, Ariel 40700, Israel; danyelchermon@gmail.com

**Keywords:** high-density lipoprotein, triglycerides, polygenic risk score, dyslipidemia, lifestyle factors

## Abstract

**Background/Objective**: Dyslipidemia significantly contributes to cardiovascular disease (CVD), with triglycerides (TG) and high-density lipoprotein cholesterol (HDL-C) as key components. While genetics play a key role in lipid levels, the interplay between genetic predisposition and modifiable lifestyle factors remains unexplored in population-based studies. We aimed to study the associations between weighted polygenic risk scores (wPRS) for TG and HDL-C, lifestyle, and metabolic factors with lipid traits and CVD. **Methods**: In this cross-sectional study, genotype, metabolic and lifestyle data from an Israeli cohort (*n* = 5584 adults) were analyzed. Individual wPRSs were constructed for TG and HDL-C based on SNPs associated with each trait. Gene-environment (lifestyle and metabolic factors) associations were evaluated by stratifying participants into high wPRS (≥90th percentile) vs. lower wPRS (<90th percentile). **Results**: High wPRSs were significantly associated with unfavorable lipid profiles (higher TG and lower HDL-C) and elevated TG/HDL-C ratios. Males and females in the high wPRS_HDL_ had 97- and 10-fold higher odds of CVD, respectively (*p* < 0.0001). Individuals with a combined high wPRS_HDL_ and wPRS_TG_ showed a 44-fold increase in CVD odds (*p* < 0.0001). Obesity (BMI > 30) and HbA1c ≥5.7% were significantly associated with elevated TG and reduced HDL-C levels, particularly in high wPRS_HDL_ and WPRS_TG_ individuals, while moderate wine (1–3 drinks/week) consumption and coffee intake (≥1 cup/day) mitigated these effects, particularly among individuals with high wPRS. **Conclusions**: Risk stratification based on genetic, lifestyle and metabolic profiles may inform personalized prevention strategies for dyslipidemia.

## 1. Introduction

Cardiovascular disease (CVD) remains the foremost cause of mortality globally, with dyslipidemia representing a pivotal contributor to its pathogenesis [1]. Dyslipidemia, characterized by aberrant lipid profiles, particularly elevated triglycerides (TG), and reduced high-density lipoprotein cholesterol (HDL-C), is a well-established cardiometabolic risk factor, often co-occurring with metabolic syndrome (MS), type 2 diabetes (T2DM), and obesity [2]. Common hypertriglyceridemia (HTG; ≥150 mg/dL) is averaging about 29.6% of the global population [3]. Each 1 mmol/L reduction in TG levels is associated with a 16% relative risk reduction in major vascular events among individuals with common HTG [4]. Conversely, HDL-C plays an anti-atherogenic role by promoting reverse cholesterol transport, facilitating cholesterol efflux from foam cell macrophages in arterial plaques and other cells to the liver for excretion [5]. Additionally, HDL-C exhibits antioxidant and anti-inflammatory properties, further contributing to its protective effects against CVD [6]. Reduced levels of HDL-C are commonly linked to dysregulated metabolic states and increased risk of coronary heart disease (CHD). Each 1 mg/dL increase in HDL-C is associated with a 2–3% reduction in CVD risk [5]. Consequently, the TG/HDL-C ratio has emerged as a promising cardiometabolic biomarker and indicator of atherogenic dyslipidemia, particularly in individuals with CHD, obesity, MS, and T2DM [7,8,9]. A recent prospective study of carbohydrate-restricted diets described “lean mass hyper-responders”—individuals who maintain markedly elevated LDL-C (≥190 mg/dL) yet exhibit very low TG and high HDL-C. Despite prolonged exposure to high LDL-C, their coronary plaque burden was not significantly greater than that of matched controls, suggesting that TG/HDL-C ratio may, in some cases, surpass LDL-C in predicting atherosclerotic risk [7]. Nevertheless, TG/HDL-C cutoffs remain undefined, as the index varies significantly by sex, body mass index (BMI), insulin resistance, ethnicity, and other comorbidities [8,10]. The genetic basis of lipid profile variability, including HDL-C, and TG levels, has been increasingly elucidated through advancements in genome-wide association studies (GWAS) [11]. Heritability estimates for blood lipids are high, ranging from 40% to 60% for HDL-C and from 35% to 48% for TG [12,13]. Numerous single-nucleotide polymorphisms (SNPs) have been identified as contributors to lipid level variability, paving the way for the development of polygenic risk scores (PRSs) [11]. PRS quantitatively aggregates the cumulative effect of multiple genetic variants on lipid traits, thereby providing a deeper understanding of an individual’s genetic predisposition to lipid abnormalities. However, while PRS are valuable tools for assessing genetic susceptibility, they are insufficient in fully explaining the heterogeneity observed in lipid levels across populations. Recent reviews highlight that most PRS studies evaluate only single gene-environment pairs and are largely derived from Caucasian cohorts, thereby restricting their portability and translational utility in diverse settings. Integrating behavioral and metabolic exposures with population-tailored scores is therefore essential to refine lipid-risk stratification [14,15,16]. This study aimed to investigate how polygenetic predisposition for HDL-C and TG levels is related to modifiable lifestyle behaviors (alcohol, coffee, smoking, SSB intake) and metabolic factors (BMI, HbA1c) to better characterize their combined associations with lipid trait variations in the general population. Rather than focusing solely on dyslipidemia, our objective was to explore common lipid variability and its modifiable contributors. By considering PRS alongside modifiable lifestyle behavioral factors, we seek to identify opportunities for early, personalized interventions, enabling individuals to optimize their lipid profiles even in the absence of overt metabolic disease.

## 2. Materials and Methods

### 2.1. Study Participants

**Figure 1:**
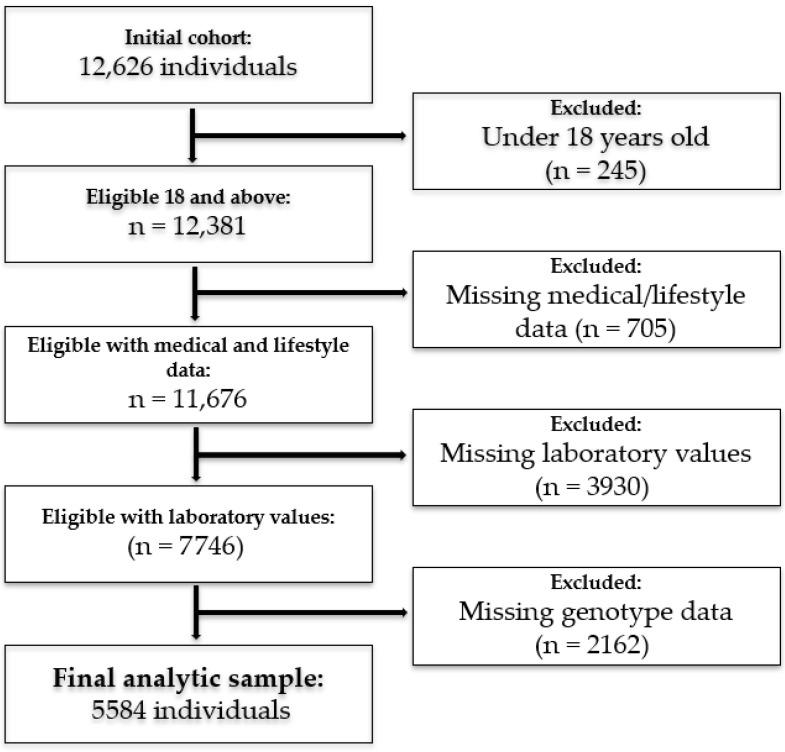
Participants selection flowchart.

Participants’ data from a community-based cohort (Jewish Israeli) were analyzed. The initial dataset included 12,626 individuals (Figure 1). After applying exclusion criteria, the final analytic cohort consisted of 5584 individuals, with 69% being female (mean age 58.0 ± 14.2 years). Exclusions included individuals under 18 years of age (*n* = 245), those with missing medical and/or lifestyle data (*n* = 705), missing laboratory values (*n* = 3930), and participants with missing genotype data (*n* = 2162). Analysis incorporated genotype profile for SNPs previously associated with lipid traits (HDL-C and TG), demographic, clinical, biochemical, and lifestyle variables. Data were obtained from the Lev Hai Genetics LTD (Registry #700068969), with all genetic information anonymized prior to analysis. The study was approved by the Ethics Committee at Ariel University (Approval #AU-HEA-RB-20220214), and all participants provided informed consent. 

### 2.2. SNPs Selection and PRS Construction

PRSs for HDL-C and TG were constructed using a clumping and thresholding approach, incorporating SNPs previously associated with each trait in large-scale GWAS. For each participant, a weighted PRS (wPRS) was calculated as the sum of the number of risk alleles at each SNP, multiplied by the respective effect size (β coefficient) derived from univariate logistic regression analyses within our cohort. To ensure quality, only SNPs in Hardy–Weinberg equilibrium were included. SNPs were excluded if they exhibited high linkage disequilibrium (LD) (R^2^ ≥ 0.8), minor allele frequency (MAF) < 0.01, or nonsignificant association with the lipid trait of interest (*p* ≥ 0.05). For wPRS_HDL_, 13 SNPs were retained out of 43 candidates for the HDL-C < 40 mg/dL threshold (applied to males): rs5882, rs3764261, rs1800961, rs2338104, rs10850219, rs1532085, rs2000813, rs2156552, rs17411031, rs2075440, rs4660293, rs7679, rs13107325. For HDL-C < 50 mg/dL threshold (applied to females), rs1800961, rs10850219, and rs2156552 were excluded due to lack of significance. For wPRS_TG_, 19 SNPs were selected from 57 initial candidates: rs2072560, rs7557067, rs157582, rs439401, rs5128, rs2068888, rs10889353, rs174546, rs2929282, rs780092, rs1260326, rs2247056, rs12678919, rs17145738, rs1495741, rs7679, rs11776767, rs2954029, rs998584. 

### 2.3. Demographic, Metabolic and Lifestyle Variables

Demographic, anthropometric, clinical, and medical data were collected through a structured, self-administered online questionnaire. Participants reported their demographic information (age, sex), anthropometric measurements (weight, height), and clinical diagnoses confirmed by a licensed physician (e.g., T2DM, CVD). Biochemical values were self-reported based on recent laboratory results from a health maintenance organization (HMO) and included HbA1c (%), total cholesterol (mg/dL), HDL-C (mg/dL), LDL-C (mg/dL), and TG (mg/dL). Smoking status was reported as either a current smoker or a non-smoker. Coffee and sugar-sweetened beverages (SSB) intakes were categorized as none or ≥1 cup per day. Wine consumption was classified as none or 1–3 drinks per week. Categorization of lifestyle exposures such as coffee, wine, smoking, and SSB consumption was based on cutoffs derived from a structured, validated questionnaire. These cutoffs were selected to reflect typical intake levels in the Israeli population and allow for adequate group comparisons. Similar categorical thresholds have been used in large-scale epidemiological studies [17,18,19]. Additional variables (e.g., high blood pressure, physical activity levels, and glucose levels) were assessed during preliminary analyses. However, none of these variables demonstrated statistically significant associations with the primary lipid outcomes (HDL-C and triglyceride levels) after adjustment for confounders. As a result, and to maintain model parsimony, these variables were excluded from the final multivariable models presented in the main analysis.

### 2.4. Statistical Analysis

Lipid traits were analyzed both as continuous variables and using clinical threshold-based categories, in accordance with the American Heart Association (AHA) guidelines; TG ≥ 150 mg/dL, HDL-C < 40 mg/dL (males) or <50 mg/dL (females) [20,21]. Due to the potential influence of menopausal status on lipid profiles, we stratified female participants by age <50 vs. age ≥ 50 years to assess whether menopause modified associations between high genetic risk and lipid outcomes. As direct menopausal status was unavailable, we used this surrogate for post-menopause, consistent with epidemiological data in Israel indicating an average menopausal age of 50–51 years. No significant differences were observed between pre- and post-menopausal females across the studied outcomes in the wPRS groups. All continuous variables were assessed for normality using Q-Q plots, histograms, and the Shapiro–Wilk test. Multivariate linear regression models were used to evaluate the association between wPRS and their respective lipid trait levels (HDL-C or TG levels in mg/dL). Logistic regression models were employed to estimate the risk of categorical outcomes adjusting for key confounders including age, sex, BMI, T2DM, and CVD medical treatment. For continuous variables not following a normal distribution, the Mann–Whitney U test was used to compare differences between the lipid thresholds groups. Categorical variables were analyzed using the Chi-square test. Continuous variables were reported as mean ± standard deviation (SD), and categorical variables as frequencies and percentages. Lipid traits (TG and HDL-C, in mg/dL) were compared across genetic risk categories—high (≥90th percentile) vs. lower (<90th percentile) wPRS [22,23]. To explore gene-environment effect modification, participants were stratified by wPRS_TG_ and wPRS_HDL_ risk groups, and within each group, mean lipid levels were compared across lifestyle and metabolic factors (e.g., obesity, HbA1c, smoking, wine, coffee, SSB). For each trait, we conducted two sets of comparisons: (1) between individuals with high vs. lower genetic risk, within each lifestyle/metabolic category, and (2) between individuals with and without the lifestyle/metabolic exposure, within each genetic risk group. A power analysis using G*Power 3.1 confirmed that the sample size (*n* = 5584) provided >95% power to detect small-to-moderate effects (Cohen’s f^2^ > 0.01) with α = 0.05. All statistical analyses were performed using Python (version 3.8), utilizing libraries such as Pandas, Scikit-learn, and Statsmodels.

## 3. Results

### 3.1. Study Characteristics

A total of 5584 adults (69.1% female, mean age 58.0 ± 14.2 years) were included in the analysis (Table 1). The majority of the cohorts were overweight or obese. Participants in the high wGRS_TG_ had significantly higher TG levels, total cholesterol (*p* < 0.0001), slightly higher LDL-C (*p* = 0.05), and a higher prevalence of SSB consumption (*p* = 0.01) compared to those in the low wGRS_TG_. Among females, those in the high wGRS_HDL_ showed significantly higher age, HbA1C, and T2DM prevalence, along with lower HDL-C and LDL-C (*p* < 0.001) compared to females in the low wGRS_HDL_ group. Similarly, males in the high wGRS_HDL_ had significantly higher HbA1c and T2DM prevalence (*p* < 0.01) and lower total cholesterol, HDL-C, and LDL-C (*p* < 0.0001) (Table 1a,b).

### 3.2. Association Between wPRS and Lipid Profiles

wPRS for TG and HDL-C showed strong associations with their corresponding lipid traits. wPRS_HDL_ was inversely associated with HDL-C levels, with each unit increase in wPRS_HDL_ corresponding to an average reduction of 4.07 mg/dL in HDL-C levels (*p* < 0.0001). Participants in the high wPRS_HDL_ had significantly lower HDL-C levels in both sexes. Mean HDL-C was 2.9 and 3.2 mg/dL higher in the females and males lower-risk group, respectively (Table 1). High wPRS_HDL_ was also associated with a significantly increased likelihood of having HDL-C below clinical thresholds. In females, those with high wPRS_HDL_ had significantly higher odds of having HDL-C < 50 mg/dL, and in males, those with high wPRS_HDL_ had significantly higher odds of having HDL-C < 40 mg/dL. For TG, participants in the high wPRS_TG_ had 34.2 mg/dL higher TG levels compared to those with lower genetic risk, (Table 1), and exhibited a significantly higher risk for TG ≥ 150 mg/dL. Each unit increase in wPRS_TG_ was associated with an average rise of 26.47 mg/dL in TG levels (*p* < 0.0001). Additionally, participants with a high wPRS_TG_ and wPRS_HDL_ had a significantly higher TG/HDL-C ratio (+0.87 mg/dL) than those at lower polygenetic risk, and were two times more likely to have a ratio of TG/HDL-C > 2 (Table 2).

### 3.3. Association Effect Between Demographic, Metabolic, and Lifestyle Factors and Lipid Trait Predisposition on Lipid Levels (mg/dL)

Lipid levels (HDL-C and TG) were significantly influenced by wPRS, with additional modulation by lifestyle and metabolic factors varying by trait and exposure (Table 3, Table 4 and Table 5).

Obesity (BMI ≥ 30): Individuals with high wPRS_TG_ and obesity had significantly elevated TG levels compared to their leaner counterparts (<0.0001). Notably, within the high wPRS_TG_, maintaining a BMI < 30 was significantly associated with lower TG levels. This reinforces that while obesity is detrimental for everyone, it may have an even greater role in exacerbating genetically driven HTG. Similarly, obesity had a significant association with HDL-C levels, particularly in genetically high-risk individuals in both sexes. Females with a high wPRS_HDL_ and obesity had significantly lower HDL-C levels than their leaner counterparts (*p* < 0.0001), reinforcing the importance of maintaining a healthy weight for HDL-C regulation. In high wPRS_HDL_ males, the association of obesity with lower HDL-C levels (mg/dL) was also significant, although slightly less pronounced than in females. In both sexes, those maintaining a BMI < 30 had markedly higher HDL-C levels, highlighting weight control as a key intervention, especially in those genetically predisposed to low HDL-C.

Sex: Mean TG levels were significantly lower for females than males in both genetic groups (Table 3).

HbA1c: High HbA1c (≥5.7%) was associated with significantly higher TG levels in both wPRS_TG_ groups (Table 3). However, the most pronounced elevations were observed in individuals with high wPRS_TG_, suggesting that poor glycemic control associates with genetically driven TG elevations, making metabolic regulation a possible relevant factor for intervention in genetically susceptible individuals (Table 3). Elevated HbA1c (≥5.7%) levels were significantly associated with lower HDL-C levels only in the low wPRS_HDL_, for both sexes (Table 4 and Table 5).

Smoking: Smoking had a differential effect depending on wPRS_TG_. Only in individuals with low wPRS_TG_, smoking significantly increased TG levels, while in individuals with high wPRS_TG_, the difference between smokers and non-smokers was not significant. This suggests that for those with high wPRS_TG_, the risk is already maximized, and smoking may not further exacerbate TG levels—a possible ceiling effect (Table 3). Smoking was significantly associated with lower HDL-C among males and low wPRS_HDL_ females, where smokers had significantly lower HDL-C levels compared to non-smokers. Notably, only in males with high wPRS_HDL_, smoking was significantly associated with lower HDL-C levels compared to males with low wPRS_HDL_. This suggests that while smoking is generally detrimental for HDL-C, it may have a more profound association in genetically predisposed males compared to females (Table 4 and Table 5).

Wine Consumption: Moderate wine consumption (1–3 drinks per week) was associated with significantly lower TG levels, with a more pronounced difference observed among individuals in high wPRS_TG_ (*p* = 0.02), whereas a smaller but statistically significant difference was also seen in the low wPRS_TG_ group (Table 3). Moderate wine consumption was associated with significantly higher HDL-C levels, within the same wPRS_HDL_ categories in males and in females and between the wPRS_HDL_ categories in males and in non-drinkers in females (Table 4 and Table 5).

Sugar-Sweetened Beverages (SSB): SSB consumption (≥1 cup/day) was significantly associated with higher TG levels and lower HDL-C levels only in individuals with low wPRS_TG_ and wPRS_HDL_.

Coffee Consumption: Moderate coffee consumption (1–3 cups per day) had no significant effect on TG levels in either genetic group. However, moderate coffee consumption was associated with higher HDL-C levels in females with high wPRS_HDL_ (*p* = 0.003).

### 3.4. wPRS Association with CVD

Among males, a significant association was observed for individuals in the highest decile of wPRS_HDL,_ exhibiting 96.64 times higher odds of CVD (β = 4.58, 95%CI 54.13–172.61), consistent with the extreme genetic burden carried by this subgroup. These estimates reflect comparisons with all other deciles and should be interpreted within the context of high cumulative polygenic risk. Similarly, females with the high wPRS_HDL_ demonstrated a significant association, with a 31.58-fold increase in CVD odds (β = 3.45, 95%CI 22.83–43.70). A composite PRS for high wPRS_HDL_ and wPRS_TG_ was also strongly associated with CVD, with individuals in this category having 10.41 times higher odds (β = 2.34, 95%CI 6.51–16.66). In contrast, wPRS_TG_ was not significantly associated with CVD. These findings suggest that individuals with both high wPRS_HDL_ and high wPRS_TG_ are at substantially increased risk for CVD (Table 6).

## 4. Discussion

This study demonstrates that genetic predisposition, quantified via polygenic risk scores for HDL-C and TG (wPRS_HDL_ and wPRS_TG_), is a major determinant of lipid profiles in a general adult population. Each unit increase in WPRS_TG_ and wPRS_HDL_ correlated with substantial lipid trait variations, reinforcing lipid traits’ heritability. The utility of wPRS in capturing lifelong genetic exposure, unlike single blood lipid measurement, supports the use of individual genetic predisposition as an important risk factor for common dyslipidemia. Furthermore, the wPRS could potentially capture CVD risk independently of blood levels through pleiotropic pathways [24]. Our wPRS, derived from known lipid-associated SNPs, reflects allele frequency and effect size patterns unique to our cohort. Other studies, for instance, by Teslovich et al., which included four SNPs overlapping with our wPRS_HDL_, found that individuals in the top HDL-C PRS quartile were four times more likely to have low HDL-C levels compared to those in the bottom quartile [11], supporting the role of higher HDL-C levels in reducing CVD mortality risk, independently of LDL-C concentration [25,26]. Mendelian randomization approaches revealed that low HDL-C levels (≤50 mg/dL) had a potentially causal inverse association with CVD risks, while no significant associations were observed at higher HDL-C levels [27].

While a TG/HDL-C ratio of< 2 is generally considered normal [28,29,30], studies in diverse populations have identified predictive thresholds ranging from approximately 1.2 to 2.6, varying by sex and cardiometabolic risk profile [9,31,32]).

To the best of our knowledge, this study is the first to explore the combined association of genetic predisposition for TG and HDL-C on TG/HDL-C ratio. Participants in the top decile for both wPRS_TG_ and wPRS_HDL_ exhibited significantly elevated ratios and were twice as likely to exceed the clinical threshold of TG/HDL-C > 2, suggesting a higher risk of cardiometabolic events in this genetically predisposed subgroup.

While genetic factors play a critical role in dyslipidemia risk, lifestyle and metabolic factors exert strong modulatory effects. Notably, our findings illustrate that the influence of wPRS on CVD risk is trait-specific. The highest wPRS_HDL_ (10th decile) was robustly associated with prevalent CVD, especially in males and to a lesser extent in females. By contrast, wPRS_TG_ alone did not significantly predict CVD risk, but the combination of both high scores conferred an exceedingly high likelihood of CVD. These high effect sizes reflect the unique risk of individuals at the extreme end of genetic liability and are biologically plausible given their distinct risk profile. Genetically driven low HDL-C appears to be a major driver to CVD susceptibility, whereas elevated TG may need to coincide with low HDL-C or other factors to substantially associate with CVD risk. This aligns with studies showing that TG-related genetic variants have modest predictive power in isolation [32], and that the combined dyslipidemia phenotype characteristics of MS yield the highest risk [8,33,34].

Sex-specific differences were observed in our study, with females consistently exhibiting lower TG levels compared to males across all genetic risk categories, aligning with previous findings identifying sex-related differences in lipid-associated loci [35,36], likely mediated by hormonal factors, with testosterone reducing HDL-C and elevating TG, whereas estrogen exerts protective effects. These observations highlight the necessity of considering sex differences into predictive models and therapeutic interventions. Glycemic control in our cohort also modified genetic risk. Individuals with HbA1c (≥5.7%) had significantly higher TG levels (mg/dL) in the high wPRS_TG_ group, and lower HDL-C levels in the low wPRS_HDL_ group, suggesting that poor glycemic control magnifies genetic susceptibility to unfavorable TG levels, whereas its effect on HDL-C may be masked in individuals with strong genetic predisposition. Obesity (BMI ≥30) emerged as a potent amplifier of genetic susceptibility in the high wPRS_TG_ group. This synergistic burden is likely mediated through an obesity-induced chronic inflammatory and insulin-resistant state, leading to disrupted lipid metabolism [37], increased fatty acids flux to the liver, enhanced intrahepatic expression of genes involved in TG biosynthesis, and impaired clearance of TG-rich lipoprotein particles, thus exacerbating HTG [38,39]. We found that moderate wine consumption was associated with an improved lipid profile among genetically predisposed participants. This included significantly lower TG levels in those with high wPRS_TG_ and higher HDL-C levels in those with high wPRS_HDL_. While evidence indicates that alcohol consumption, including wine association with TG and HDL-C levels can be influenced by genetic individual responses [40,41], a large population-based study found HDL-C-related SNPs (APOA5, CETP, LIPC, LPL) were independently associated with HDL-C levels, but no significant gene–alcohol interactions were detected, suggesting the HDL-C-raising effects of alcohol may occur independently of genotype [42], possibly through flavonoids-mediated nitric oxide bioavailability, improved endothelial function, and reverse cholesterol transport [43]. Similarly, our findings show that moderate coffee intake (1–3 cups/day) was linked to higher HDL-C levels only in predisposed females, suggesting a sex-specific gene–lifestyle interplay. Several studies reported that moderate coffee intake of 3–5 daily cups might offer cardioprotective benefits compared to abstention, though findings regarding lipid profiles are mixed [44,45]. A study among Taiwanese adults reported a significant increase in HDL-C levels with black coffee consumption of 4.07 mg/dL with 1–4 cups/week (*p* = 0.0007) and 4.13 mg/dL with ≥5 cups/week (*p* = 0.0008), compared to non-consumers [46]. Similarly, a Taiwan Biobank study (N > 9000) found that coffee consumption was positively associated with HDL-C levels in females but not in males. Importantly, this effect remained after adjusting for relevant genetic polymorphisms, suggesting that both genetic and non-genetic mechanisms are involved [47]. By contrast, a meta-analysis showed no significant effect of coffee on HDL-C levels, despite increases in total cholesterol, TG, and LDL-C (mg/dL) [48]. These discrepancies may stem from variations in study design, population demographics, coffee preparation methods, and the presence of additives. Notably, certain components in coffee, particularly the diterpenes cafestol and kahweol, are known to alter lipid metabolism, possibly lowering HDL-C via increased cholesterol ester transfer protein (CETP) activity and altered lecithin–cholesterol acyltransferase (LCAT)/phospholipid transfer protein (PLTP) functions [49]. However, such effects are typically observed at high diterpene doses equivalent to 10–20 cups of boiled coffee per day, far exceeding our study’s exposure. Smoking was associated with higher TG levels only among participants with low wPRS_TG_, indicating a possible ceiling effect, where genetically driven HTG is already maximized and not further aggravated by smoking. For HDL-C levels, smoking reduced levels only in genetically predisposed males, reflecting hormonal differences in lipid metabolism. Smoking adversely affects lipid metabolism through multiple mechanisms, including oxidative modifications of HDL-C particles and reduced activity of key lipid-transport enzymes, such as LCAT, CETP, and hepatic lipase [50]. These findings align with studies on gene–smoking interactions and Mendelian randomization analyses showing amplified cardiometabolic risk in genetically susceptible smokers [51]. SSB consumption was associated with adverse lipid profiles only in low wPRS_TG_ and wPRS_HDL_. This pattern may reflect a possible ceiling effect, where lifestyle behaviors may have diminished association in genetically high-risk individuals. These findings mirror previous large cohort studies showing no gene×SSB interactions [52].

Our study, while comprehensive, is not without limitations. While our stratified descriptive analyses revealed noteworthy differences across genetic risk groups, these results must be interpreted cautiously. The cross-sectional design precludes the establishment of causality between PRS, lifestyle factors, and lipid levels or CVD outcomes. While the large sample size allowed for high statistical power, many comparisons yielded extremely small *p*-values. These results should be interpreted in light of effect sizes and confidence intervals rather than statistical significance alone. Furthermore, in certain stratified analyses, such as CVD risk, the number of events was relatively small, which may have led to inflated odds ratios due to sparse data. These findings should therefore be interpreted with caution and validated in future studies. Additionally, reliance on self-reported data for lifestyle habits, biochemical parameters, and clinical diagnoses may introduce recall bias or inaccuracies. Nevertheless, our findings are derived from an Israeli cohort, which exhibited a high prevalence of overweight/obesity, potentially limiting generalizability to other ethnic populations or populations with different metabolic profiles; validation in diverse cohorts is warranted. Despite these limitations, our findings have potential implications for clinical practice: incorporating PRS, particularly for HDL-C and combined TG/HDL-C, alongside traditional risk factors and lifestyle assessment could refine CVD risk stratification. This study highlights specific factors such as obesity and poor glycemic control as potent amplifiers of genetic risk, suggesting that individuals with high genetic susceptibility may benefit most from aggressive management of these modifiable factors. Conversely, the potential mitigating effects observed for moderate wine or coffee intake in high-risk groups, while requiring cautious interpretation and further study, hint at personalized lifestyle counseling opportunities. While PRS are not yet standard clinical tools, this research supports their potential utility in identifying high-risk individuals who warrant intensified preventive efforts.

## 5. Conclusions

Integrating wPRSs for lipid traits with demographic, metabolic, and lifestyle data refines the need for stratification of dyslipidemia risk. This combined approach may help identify subgroups who are more responsive to lifestyle interventions and optimize precision strategies for reducing lipid-related cardiovascular morbidity. Future longitudinal and interventional studies are needed to further evaluate the clinical utility of this approach in guiding precision lipid management.

## Figures and Tables

**Table 1:** (**a**) Descriptive characteristics of study participants stratified by wPRS_TG_. (**b**) Descriptive characteristics of study participants stratified by wPRS_HDL_.

(a)										
Variable		All Population		wPRS_TG_ < 90			wPRS_TG_ ≥ 90		*p*-Value	
Sex (female) *n*, (%)		3861 (69.1)		3461 (68.9)			400 (71.7)		0.18	
Age (years) (mean ± SD)		58.0 ± 14.2		58.0 ± 14.2			58.1 ± 14.0		0.83	
BMI (kg/m^2^) (mean ± SD)		31.3 ± 5.8		31.3 ± 5.8			30.9 ± 5.1		0.21	
HbA1C (%) (mean ± SD)		5.1 ± 2.7		5.1 ± 2.5			5.2 ± 3.6		0.76	
Total Cholesterol (mg/dL)		183.4 ± 48.6		182.5 ± 48.1			191.9 ± 51.9		<0.0001	
HDL-C (mg/dL)		52.9 ± 12.5		52.9 ± 12.5			52.5 ± 11.9		0.47	
LDL-C (mg/dL)		109.2 ± 37.2		109.0 ± 36.5			110.9 ± 43.1		0.05	
TG (mg/dL)		130.5 ± 65.8		127.1 ± 63.2			161.3 ± 79.4		<0.0001	
T2DM *n*, (%)		471 (8.4)		420 (8.4)			51 (9.1)		0.58	
CVD *n*, (%)		486 (8.7)		437 (8.7)			49 (8.8)		0.94	
Smoking *n*, (%)		507 (9.1)		458 (9.1%)			49 (8.8)		0.85	
SSB (≥1 cup/day) *n*, (%)		930 (16.7%)		817 (16.3%)			113 (20.3)		0.01	
Coffee (≥1 cup/day) *n*, (%)		4234 (75.8)		3806 (75.7)			428 (76.7)		0.64	
Wine (1–3 drinks/week) *n*, (%)		1721 (30.8)		1555 (30.9)			166 (29.7)		0.47	
(**b**)										
**Variable**	**wPRS_HDL_ < 90**		**wPRS_HDL_ ≥ 90**		***p*-Value**	**wPRS_HDL_ < 90**		**wPRS_HDL_ ≥ 90**		***p*-Value**
	**Females**					**Males**				
Age (years) (mean ± SD)	57.2 ± 13.7		61.1 ± 14.1		<0.0001	57.1 ± 15.0		64.3 ± 12.7		<0.0001
BMI (kg/m^2^) (mean ± SD)	30.9 ± 5.8		31.3 ± 6.0		0.09	31.8 ± 5.5		32.3 ± 5.6		0.12
HbA1C (%) (mean ± SD)	5.1 ± 2.7		5.3 ± 3.3		<0.001	5.1 ± 2.5		5.3 ± 2.1		<0.001
Total Cholesterol (mg/dL)	190.8 ± 46.9		179.0 ± 49.9		<0.0001	174.6 ± 48.1		156.7 ± 47.0		<0.0001
HDL-C (mg/dL)	56.7 ± 12.0		53.8 ± 11.8		<0.0001	45.9 ± 9.8		42.7 ± 9.3		<0.0001
LDL-C (mg/dL)	113.6 ± 35.7		104.3 ± 36.7		<0.0001	106.3 ± 38.0		88.6 ± 39.4		<0.0001
TG (mg/dL)	124.5 ± 60.7		127.9 ± 60.5		0.08	142.0 ± 74.2		145.9 ± 75.9		0.39
T2DM *n*, (%)	205 (6.2)		59 (10.4)		<0.001	147 (10.8)		60 (16.5)		<0.01
CVD *n*, (%)	55 (1.7)		206 (36.2)		<0.0001	18 (1.3)		207 (56.9)		<0.0001
Smoking *n*, (%)	299 (9.1)		45 (7.9)		0.40	137 (10.1)		26 (7.1)		0.10
SSB (≥1 cup/day) *n*, (%)	481 (14.6)		89 (15.6)		0.56	290 (21.3)		70 (19.2)		0.42
Coffee (≥1 cup/day) *n*, (%)	2494 (75.8)		420 (73.8)		0.32	1028 (75.6)		292 (80.2)		0.07
Wine (1–3 drinks/week) *n*, (%)	909 (27.6)		141 (24.8)		0.31	522 (38.4)		149 (40.9)		0.68

**Table 2:** Risk for corresponding lipid thresholds by wPRS (≥90th percentile).

wPRS Category *	β	OR	95%CI	*p*-Value
wPRS_HDL_ males	0.572	1.77	1.39–2.26	<0.0001
wPRS_HDL_ females	0.419	1.52	1.26–1.84	<0.0001
wPRS_TG_	0.946	2.58	2.15–3.09	<0.0001
Combined wPRS_HDL_ and wPRS_TG_	0.692	2.00	1.26–3.17	0.003

OR, odds ratio; wPRS, weighted polygenic risk score; * (≥90th percentile vs. <90 percentile). Adjusted for age, sex, BMI, and T2DM (wPRSHDL was not adjusted for sex due to stratification by sex).

**Table 3:** TG levels stratified by genetic risk and lifestyle/metabolic variables.

Variable	TG Levels (Mean ± SD) in High * wPRS_TG_	TG Levels (Mean ± SD) in Low * wPRS_TG_	*p*-Value (a)
Sex			
Male	172.46 ± 80.00	139.82 ± 73.32	*p* < 0.0001
Female	156.77 ± 78.87	121.28 ± 57.10	*p* < 0.0001
*p*-value (b) within wPRS_TG_	0.008	*p* < 0.0001	
Wine			
Non-drinkers	166.43 ± 81.23	129.70 ± 64.35	*p* < 0.0001
1–3 drinks/week	148.86 ± 74.37	121.56 ± 60.13	*p* < 0.0001
*p*-value (b) within wPRS_TG_	0.02	*p* < 0.0001	
Coffee			
Non-consumers	166.62 ± 78.42	127.56 ± 63.75	*p* < 0.0001
1–3 cups/day	161.75 ± 77.70	127.92 ± 63.96	*p* < 0.0001
*p*-value (b) within wPRS_TG_	0.41	0.94	
Obesity			
BMI < 30	140.85 ± 68.94	114.82 ± 58.64	*p* < 0.0001
BMI ≥ 30	178.98 ± 83.72	136.96 ± 64.97	*p* < 0.0001
*p*-value (b) within wPRS_TG_	*p* < 0.0001	*p* < 0.0001	
Smoking			
Non-smoker	159.23 ± 75.03	126.14 ± 62.41	*p* < 0.0001
Smoker	181.76 ± 114.73	136.19 ± 69.82	*p* < 0.01
*p*-value (b) within wPRS_TG_	0.55	0.002	
SSB			
None	160.83 ± 82.55	125.29 ± 61.50	*p* < 0.0001
≥1 cup/day	162.72 ± 66.05	136.14 ± 70.56	*p* < 0.0001
*p*-value (b) within wPRS_TG_	0.33	0.01	
HbA1c			
<5.7%	149.98 ± 74.05	118.12 ± 57.41	*p* < 0.0001
≥5.7%	182.80 ± 84.99	143.42 ± 69.67	*p* < 0.0001
*p*-value (b) within wPRS_TG_	*p* < 0.0001	*p* < 0.0001	

(a) Compares mean TG levels between wPRS_TG_ categories (low vs. high). (b) Compares mean TG levels between lifestyle/metabolic categories within wPRS_TG_ category. SSB; Sugar-Sweetened Beverages * high = ≥90th percentile, low = <90th percentile.

**Table 4:** HDL-C levels stratified by genetic risk and lifestyle/metabolic variables in females.

Variable	HDL-C Levels (Mean ± SD) in High * wPRS_HDL_	HDL-C Levels (Mean ± SD) in Low * wPRS_HDL_	*p*-Value (a)
Wine			
Non-drinkers	52.86 ± 11.98	56.18 ± 11.73	*p* < 0.0001
1–3 drinks/week	56.40 ± 10.46	57.59 ± 12.32	0.61
*p*-value (b) within wPRS_HDL_	<0.001	0.01	
Coffee			
Non-consumers	51.52 ± 12.29	55.98 ± 11.66	*p* < 0.0001
1–3 cups/day	54.38 ± 11.56	56.92 ± 12.18	0.001
*p*-value (b) within wPRS_HDL_	0.003	0.23	
Obesity			
BMI < 30	57.28 ± 11.90	59.14 ± 12.36	0.06
BMI ≥ 30	51.12 ± 11.07	54.56 ± 11.26	*p* < 0.0001
*p*-value (b) within wPRS_HDL_	*p* < 0.0001	*p* < 0.0001	
Smoking			
Non-smoker	53.93 ± 11.82	57.03 ± 11.96	*p* < 0.0001
Smoker	52.24 ± 11.97	53.82 ± 12.15	0.44
*p*-value (b) within wPRS_HDL_	0.33	*p* < 0.0001	
SSB			
None	53.87 ± 11.83	57.18 ± 12.17	*p* < 0.0001
≥1 cup/day	53.39 ± 11.87	54.12 ± 10.71	0.54
*p*-value (b) within wPRS_HDL_	0.77	*p* < 0.0001	
HbA1c			
<5.7%	53.86 ± 11.73	57.62 ± 12.11	*p* < 0.0001
≥5.7%	53.68 ± 12.00	54.92 ± 11.60	0.20
*p*-value (b) within wPRS_HDL_	0.92	*p* < 0.0001	

(a) Compares mean HDL-C levels between wPRS_HDL_ categories. (b) Compares mean HDL-C levels within each wPRS_HDL_ category. SSB; Sugar-Sweetened Beverages. * high = ≥90th percentile, low = <90th percentile.

**Table 5:** HDL-C levels stratified by genetic risk and lifestyle/metabolic variables in males.

Variable	HDL-C Levels (Mean ± SD) in High * wPRS_HDL_	HDL-C Levels (Mean ± SD) in Low * wPRS_HDL_	*p*-Value (a)
Wine			
Non-drinkers	43.60 ± 8.82	44.50 ± 9.42	*p* < 0.0001
1–3 drinks/week	44.96 ± 9.67	46.14 ± 9.91	0.006
*p*-value (b) within wPRS_HDL_	0.03	0.007	
Coffee			
Non-consumers	45.62 ± 11.75	45.61 ± 9.93	0.02
1–3 cups/day	43.67 ± 7.98	45.29 ± 10.02	0.0001
*p*-value (b) within wPRS_HDL_	0.98	0.58	
Obesity			
BMI < 30	45.83 ± 10.55	47.82 ± 10.82	<0.001
BMI ≥ 30	42.89 ± 7.55	43.65 ± 8.69	*p* < 0.0001
*p*-value (b) within wPRS_HDL_	0.002	*p* < 0.0001	
Smoking			
Non-smoker	44.36 ± 8.89	45.57 ± 9.85	*p* < 0.0001
Smoker	42.61 ± 10.72	42.97 ± 9.21	0.004
*p*-value (b) within wPRS_HDL_	0.01	<0.01	
SSB			
None	44.63 ± 9.04	45.69 ± 10.06	*p* < 0.0001
≥1 cup/day	42.98 ± 9.15	43.86 ± 8.65	0.03
*p*-value (b) within wPRS_HDL_	0.43	0.002	
HbA1c			
<5.7%	44.79 ± 9.85	45.89 ± 9.58	*p* < 0.0001
≥5.7%	43.18 ± 7.64	44.43 ± 10.13	0.00076
*p*-value (b) within wPRS_HDL_	0.30	0.008	

(a) Compares mean TG levels between wPRSTG categories (low vs. high). (b) Compares mean TG levels between lifestyle/metabolic categories within wPRSTG category. SSB; Sugar-Sweetened Beverages. * high = ≥90th percentile, low = <90th percentile.

**Table 6:** Risk for CVD by wPRS Category.

wPRS Category *	CVD *n*, (%)	β	OR	95%CI	*p*-Value
wPRS_HDL_ males	207 (56.9)	4.581	96.64	54.13–172.61	*p* < 0.0001
wPRS_HDL_ females	206 (36.2)	3.453	31.58	22.83–43.70	*p* < 0.0001
High wPRS_TG_	49 (8.8)	0.023	1.023	0.74–1.4	0.89
Combined high wPRS_HDL_ and wPRS_TG_	40 (42.6)	2.343	10.41	6.51–16.66	*p* < 0.0001

OR, odds ratio; wPRS, weighted polygenic risk score; * high = ≥90th percentile vs. low = <90th percentile. Adjusted for age, sex, BMI, T2DM, and medical treatment (wPRS_HDL_ was not adjusted for sex due to stratification by sex).

## Data Availability

The data are not publicly available due to privacy reasons.
